# Thrombolytic Therapy in the Oldest Old: Successful Alteplase Administration in a 105-Year-Old Female With Ischemic Stroke

**DOI:** 10.7759/cureus.19346

**Published:** 2021-11-08

**Authors:** Sambhawana Bhandari, Maun R Baral, Christian Espana Schmidt

**Affiliations:** 1 Internal Medicine, Danbury Hospital, Nuvance Health, Danbury, USA

**Keywords:** alteplase, tissue plasminogen activator (tpa), 105 year old and alteplase, tpa in elderly, alteplase in elderly, stroke in elderly, oldest old and thrombolysis

## Abstract

The elderly have a higher incidence of ischemic stroke along with higher mortality and morbidity compared to their younger counterparts. However, though studies have included people >85 or 90 years of age, there haven’t been reports of many individuals above 100 years of age. Among the oldest old, the oldest age studied for alteplase administration is possibly 101 years.

We present a case of a 105-year-old woman with a medical history of a cerebrovascular accident in 2018 for which she received thrombolytic therapy with residual slurred speech and mild dysphagia. She was brought in from her nursing facility for worsening of slurred speech and right facial droop that began two hours before presentation to the emergency department. She was found to have aphasia, left-sided gaze preference, and upper motor neuron dysfunction of her right arm associated with drift. Her National Institutes of Health Stroke Scale (NIHSS) score at presentation was 17. An urgent CT scan of the head was performed that did not show any evidence of acute bleeding. CT angiogram of brain and neck was unremarkable with no evidence of high-grade stenosis, aneurysm, occlusion, or dissection. With the negative CT findings, the patient received thrombolytic therapy with alteplase*. *She was monitored in the intensive care unit for 24 hours after alteplase administration with neurological checks every one hour. The repeated CT head after 24 hours was negative for any hemorrhage. She was discharged after two days of hospital admission and her NIHSS score on the day of discharge was 4. Our patient was 105 years old, which is arguably one of the oldest ages to receive alteplase. Her NIHSS score improved considerably and she did not suffer from hemorrhagic complications.

## Introduction

The elderly have a higher incidence of ischemic stroke along with higher mortality and morbidity in comparison to younger counterparts. However, rates of symptomatic hemorrhage after thrombolysis haven't been seen to be significantly different from that of the younger age group [1]. The majority of patients aged >90 years benefited from treatment with alteplase within the three-hour treatment time frame [2]. Among the oldest old (>85 years), the oldest age studied for alteplase administration is likely 101 years [3].

## Case presentation

We present a case of a 105-year-old female with a medical history of a cerebrovascular accident in 2018 for which she received thrombolytic therapy with residual slight slurring of speech and mild dysphagia. Other medical history included Barrett's esophagus, hypertension, history of seizures, and gastroesophageal reflux disease (GERD). Surgical history included a history of gastric carcinoma s/p Billroth II and heart block s/p pacemaker. She also had previous admissions due to acute metabolic encephalopathy secondary to urinary tract infection.

She was brought in from her nursing facility for worsening slurred speech and new onset of right-sided facial droop. Due to her impaired speech, it was very difficult to get a meaningful history from her and most of the history was obtained from her daughter, nursing facility records, and her aid. As per nursing facility records, she was taking Plavix for a prior history of stroke but wasn’t on any other blood thinners. According to her aide, at baseline, she had minimally slurred speech with dysphagia and was able to move all extremities well. The patient was last seen to be at her baseline by her aide about two hours prior. She immediately alerted the emergency medical services (EMS) after she noticed the patient’s slurred speech and right facial droop. The patient did not have any recent falls or witnessed convulsive activity.

On presentation to the emergency room, she was hemodynamically stable, afebrile, heart rate was 90 beats/min, blood pressure was 120-140 mm Hg over 60-70 mm Hg, respiratory rate was 20 breaths/minute, and saturation was 90% on room air. On examination, she was alert, orientation couldn't be assessed due to aphasia. She had absent blink to threat on the right and intact on the left. She had a left gaze preference. Right-sided facial asymmetry was noticed with a grimace. Protrusion of tongue couldn't be assessed as she refused. She was able to lift and sustain the right arm against gravity but there was downward drift. She was able to lift and sustain the left arm as well. She didn't withdraw to noxious stimuli in the right arm and showed purposeful withdrawal to noxious stimuli on the left. Plantar reflex was mute bilaterally. National Institutes of Health Stroke Scale (NIHSS) score was noted to be 17.

An emergent CT scan of the head didn't show any evidence of acute bleed. CT angiogram of the brain and neck was unremarkable without any evidence of high-grade stenosis, aneurysm, occlusion, or dissection. Blood work showed a white blood cell count of 11.3/L, hemoglobin of 14 g/dL, platelets of 310/L, sodium of 135 mEq/L, potassium of 5.4 mEq/L, creatinine of 0.5mg/dL, glucose of 147 mg/dL, prothrombin time/international normalized ratio (PT/INR) of 12.3/0.94, and a positive rapid COVID-19 test. As per the daughter, the patient was vaccinated with two doses of COVID-19 vaccine two weeks ago and didn't have any sick contacts in the last month. Her 24 hours aides were tested weekly for COVID-19 and were negative.

Immediate neurology consultation was done by the emergency department staff who concluded that the patient's aphasia, left gaze preference, and upper motor neuron dysfunction of the right hemicorpus was likely due to an acute ischemic stroke in the left middle cerebral artery distribution. These recommendations were conveyed to the patient's daughter over the phone and discussion was done about the risks and benefits of alteplase therapy. After the discussion, a mutual decision was made to administer alteplase within a few minutes of her presentation to the emergency room.

She was admitted to the intensive care unit for close monitoring of vital signs and every one-hour neuro checks for 24 hours. Plavix was held for 24 hours after alteplase. An echocardiogram showed a normal ejection fraction of 60%, normal systolic function with no regional wall motion abnormalities, valvular abnormalities, or vegetations. Repeat CT head 24 hours later was negative for any hemorrhage but showed mild microvascular disease and diffuse age-related involutional changes seen in prior CT scans. Plavix was then resumed for secondary stroke prevention.

In terms of her positive rapid COVID-19 test, she remained asymptomatic without any documented hypoxia throughout the hospital stay. No other therapies were indicated then except for prophylactic anticoagulation. This was evaluated by the infectious disease department and it was recommended that the patient remain isolated in the facility for up to 14 days from the day of the positive test. She was discharged back to her facility after being seen by physical therapy, speech and swallow specialists, and her NIHSS score on the day of discharge was 4.

## Discussion

Older age is the most common non-modifiable risk factor for stroke, which is a common cause of death and disability worldwide. As per American Community Survey reports (2018), in 2015, among the 7.3 billion people estimated worldwide, 617.1 million (9%) were aged 65 years and older. By 2030, the older population will be about one billion (12% of the projected total world population) [4]. With this trend in life expectancy, the rates of stroke and its burden on the quality of life will only continue to increase.

Intravenous alteplase (tissue plasminogen activator) is a widely used and approved treatment for acute ischemic stroke. However, clinical trial evidence for use of intravenous alteplase is limited for the elderly although there has been growing interest and analysis proving efficacy and both long and short-term safety [3].

The 2013 American Heart Association/American Stroke Association (AHA/ASA) guidelines for the early management of patients with acute ischemic stroke recommended intravenous alteplase as early as possible for eligible adult stroke patients who may be treated within three hours of symptom onset. The effectiveness is not well established for patients >80 years of age for treatment in the time period of three to 4.5 hours after symptom onset [5]. Our patient received intravenous alteplase within three hours of her symptom onset and didn’t have any immediate side effects including hemorrhagic transformation.

There also have been studies that show no significant difference in the quality of life, neurological recovery, and functional status between the elderly and young age group post thrombolytic treatment [6]. This is very important to consider as stroke can significantly impact the quality of life in the elderly.

Although studies have included people >85 years of age, there haven’t been reports of many individuals above the age of 100 years. Our patient was 105 years of age, arguably one of the oldest ages receiving intravenous tissue plasminogen activator. Her NIHSS score also improved considerably. She thankfully didn’t suffer from hemorrhagic complications in the CT head done 24 hours after either. She was able to be discharged back to her facility safely after about four days of hospitalization. This was also her second ischemic stroke in the past three years and she received intravenous alteplase both times and had minimal residual weakness after. We however don’t have long-term follow-up data on the patient.

She also interestingly tested positive for COVID-19 even though she received both doses of vaccine at least two weeks prior to her positive test. She was asymptomatic in terms of respiratory complaints and her oxygen saturation was well above 90% in room air. As we all are aware, COVID-19 is known to be a very hypercoagulable state due to an increase in prothrombotic factors and inflammatory cytokines [7]. As such, it has been very commonly associated with ischemic stroke. Although stroke is usually seen in cases of symptomatic COVID-19 infection, there have been many case reports with asymptomatic ones [8,9]. There is no way to be certain that COVID-19 infection was the cause of her stroke; however, there is a strong temporal relationship between COVID-19 positivity and stroke in this scenario.

## Conclusions

Tissue plasminogen activator administration still needs to be better studied in the oldest old. However, in limited studies done, it has shown to have similar morbidity and mortality rates in comparison to the younger age group. Thus, intravenous thrombolysis should be considered for all very elderly patients with ischemic stroke and should be further individualized based on the associated risks, benefits, contraindications, and comorbidities as it can significantly impact their quality of life.
